# 3,3-Bis[(4-chloro­phen­yl)sulfan­yl]-1-methyl­piperidin-2-one

**DOI:** 10.1107/S1600536810024347

**Published:** 2010-06-30

**Authors:** Julio Zukerman-Schpector, Carlos A. De Simone, Paulo R. Olivato, Carlos R. Cerqueira, Jean M. M. Santos, Edward R. T. Tiekink

**Affiliations:** a Department of Chemistry, Universidade Federal de São Carlos, 13565-905 São Carlos, SP, Brazil; b Instituto de Química e Biotecnologia, Universidade Federal de Alagoas, 57072-970 Maceió, AL, Brazil; c Chemistry Institute, Universidade de São Paulo, 05508-000 São Paulo-SP, Brazil; d Department of Chemistry, University of Malaya, 50603 Kuala Lumpur, Malaysia

## Abstract

The piperidone ring in the title compound, C_18_H_17_Cl_2_NOS_2_, has a distorted half-chair conformation. The S-bound benzene rings are approximately perpendicular to and splayed out of the mean plane through the piperidone ring [dihedral angles = 71.86 (13) and 46.94 (11)°]. In the crystal, C—H⋯O inter­actions link the mol­ecules into [010] supra­molecular chains with a helical topology. C—H⋯Cl and C—H⋯π inter­actions are also present.

## Related literature

For background to β-thiocarbonyl compounds, see: Vinhato (2007 ▶); Olivato *et al.* (2009 ▶). For related structures, see: Zukerman-Schpector *et al.* (2006 ▶, 2008 ▶). For ring conformational analysis, see: Cremer & Pople (1975 ▶). For further synthetic details, see: Hashmat & McDermott (2002 ▶); Zoretic & Soja (1976 ▶).
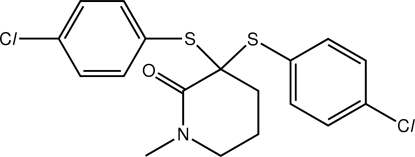


## Experimental

### 

#### Crystal data

C_18_H_17_Cl_2_NOS_2_
*M* *_r_* = 398.37
Monoclinic, 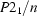

*a* = 8.0313 (2) Å
*b* = 9.7460 (2) Å
*c* = 24.2623 (7) Å
β = 94.0767 (12)°
*V* = 1894.28 (8) Å^3^
*Z* = 4
Mo *K*α radiation
μ = 0.57 mm^−1^
*T* = 290 K
0.33 × 0.30 × 0.29 mm

#### Data collection

Nonius KappaCCD diffractometer
Absorption correction: multi-scan (*SADABS*; Sheldrick, 1996 ▶) *T*_min_ = 0.82, *T*_max_ = 0.85
12888 measured reflections
3288 independent reflections
2778 reflections with *I* > 2σ(*I*)
*R*_int_ = 0.049

#### Refinement

*R*[*F* ^2^ > 2σ(*F* ^2^)] = 0.043
*wR*(*F* ^2^) = 0.121
*S* = 1.05
3288 reflections
218 parameters
H-atom parameters constrained
Δρ_max_ = 0.37 e Å^−3^
Δρ_min_ = −0.38 e Å^−3^

### 

Data collection: *COLLECT* (Nonius, 1999 ▶); cell refinement: *SCALEPACK* (Otwinowski & Minor, 1997 ▶); data reduction: *DENZO* (Otwinowski & Minor, 1997 ▶) and *SCALEPACK*; program(s) used to solve structure: *SIR97* (Altomare *et al.*, 1999 ▶); program(s) used to refine structure: *SHELXL97* (Sheldrick, 2008 ▶); molecular graphics: *ORTEP-3* (Farrugia, 1997 ▶) and *DIAMOND* (Brandenburg, 2006 ▶); software used to prepare material for publication: *WinGX* (Farrugia, 1999 ▶).

## Supplementary Material

Crystal structure: contains datablocks global, I. DOI: 10.1107/S1600536810024347/hb5512sup1.cif

Structure factors: contains datablocks I. DOI: 10.1107/S1600536810024347/hb5512Isup2.hkl

Additional supplementary materials:  crystallographic information; 3D view; checkCIF report

## Figures and Tables

**Table 1:** Hydrogen-bond geometry (Å, °) *Cg*1 is the centroid of the C7–C12.

*D*—H⋯*A*	*D*—H	H⋯*A*	*D*⋯*A*	*D*—H⋯*A*
C9—H9⋯O1^i^	0.93	2.32	3.218 (3)	164
C11—H11⋯Cl2^ii^	0.93	2.83	3.708 (3)	157
C19—H19a⋯*Cg*1^iii^	0.96	2.95	3.676 (3)	133

Symmetry codes: (i) 
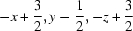
; (ii) 
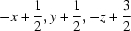
; (iii) 
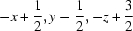
.

**Fig. 1.**
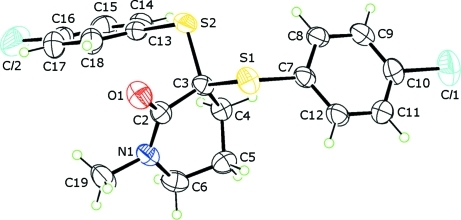
The molecular structure of (I) showing displacement ellipsoids at the 35% probability level (arbitrary spheres for the H atoms).

**Fig. 2.**
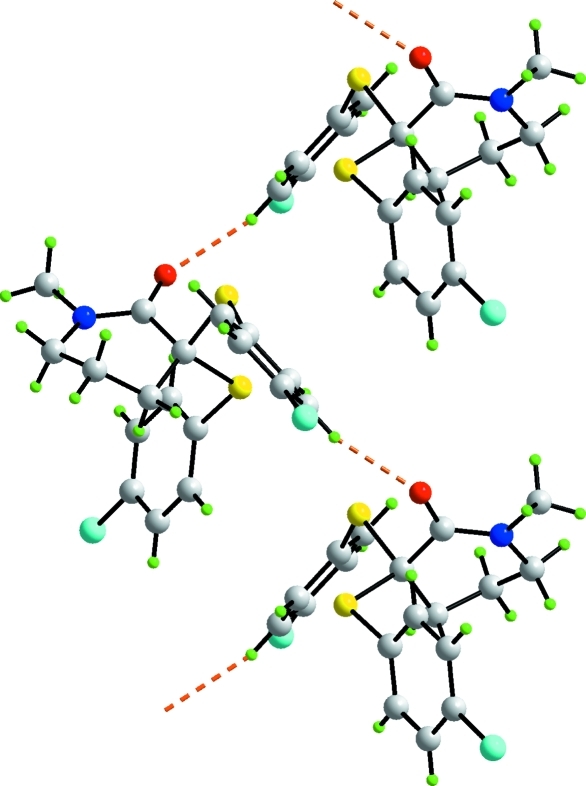
Supramolecular chain in (I) mediated by C–H···O interactions (orange dashed lines). The chain with helical topology is aligned along the b axis.

## supplementary crystallographic information

### Comment

As part of our on-going research on the conformational and electronic interactions in β-thio-carbonyl compounds, *e.g. N*,*N*-diethyl-2-[(4'-substituted) phenylthio]acetamides, *N*-methoxy-*N*-methyl-2-[(4'-substituted) phenylthio]propanamides, and 1-methyl-3-phenylsulfonyl-2-piperidone, utilizing spectroscopic, theoretical and X-ray diffraction methods (Vinhato, 2007; Olivato *et al.*, 2009; Zukerman-Schpector *et al.* 2008), the title compound, (I), was synthesized and its crystal structure determined.

In (I), Fig. 1, the piperidone ring has a distorted half-chair conformation: the ring-puckering parameters are q_2_ = 0.453 (2) Å, q_3_ = -0.271 (2) Å, QT = 0.528 (3) Å, φ_2_ = 37.4 (3) ° (Cremer & Pople, 1975). While the S2-bound benzene ring is orientated to be almost perpendicular to the plane through the piperidone ring [dihedral angle = 71.86 (13) °], the S1-bond benzene ring is somewhat splayed with respect to the other rings, forming dihedral angles of 46.94 (11) and 61.68 (13) ° with those through the piperidone and S2-bound benzene rings, respectively.

Supramolecular helical chains aligned along the *b* axis dominate the crystal packing, Fig. 2 and Table 1, and these are sustained in the crystal structure by C–H···Cl and C–H···π interactions, Table 1.

### Experimental

Firstly, 4-chlorothiophenol (5.8 g, 40 mmol) was reacted with bromine (1.1 ml, 40 mmol) in dichloromethane (250 ml) on hydrated silica gel support (25 g of SiO_2_ and 12 ml of water) to give 4-chlorophenyl disulfide (5.3 g, yield = 93%). A yellow solid was obtained after filtration and evaporation without further purification (Hashmat & McDermott, 2002). 1-Methyl-2-piperidinone (2.0 g, 18 mmol) was added dropwise to a cooled (195 K) solution of hexamethylphosphoramide (HMPA) (3.3 ml, 18 mmol), diisopropylamine (2.6 ml, 18 mmol) and butyllithium (11.5 ml, 18 mmol) in THF (60 ml). After 20 minutes, 4-chlorophenyl disulfide (5.3 g, 18 mmol) dissolved in THF (10 ml) was added dropwise to the enolate solution (Zoretic & Soja, 1976). After stirring for 3 h at 195 K, water (80 ml) was added at room temperature and extraction with chloroform was performed. The organic layer was dried over anhydrous sodium sulfate. After evaporation of solvent, a crude solid was obtained. Purification through flash chromatography with a solution of hexane and ethyl acetate in a 7:3 ratio give the pure product (2.8 g, yield = 35%). Irregular lumps of (I) were obtained by vapour diffusion of *n*-hexane into a chloroform solution held at 283 K; m.p. 372–373 K. IR (cm^-1^): ν(C=O) 1663. NMR (CDCl_3_, p.p.m.): δ 1.93–1.95 (2*H*, m), 1.97–1.99 (2*H*, m), 2.91 (3*H*, s), 3.21 (2*H*, triplet, J = 6.0 Hz), 7.31–7.33 (4*H*, m, Aryl-H), 7.55–7.57 (4*H*, m, Aryl-H). Analysis found: C 54.33, H 4.30, N 3.39%. C_18_H_17_OCl_2_NS_2_ requires: C 54.27, H 4.30, N 3.52%.

### Refinement

The H atoms were geometrically placed (C–H = 0.93–0.97 Å) and refined as riding with *U_iso_*(H) = 1.2–1.5*U_eq_*(C).

### Crystal data

**Table:**

C_18_H_17_Cl_2_NOS_2_	*F*(000) = 824
*M**_r_* = 398.37	*D*_x_ = 1.397 Mg m^−^^3^
Monoclinic, *P*2_1_/*n*	Mo *K*α radiation, λ = 0.71073 Å
Hall symbol: -P 2yn	Cell parameters from 10679 reflections
*a* = 8.0313 (2) Å	θ = 2.9–27.5°
*b* = 9.7460 (2) Å	µ = 0.57 mm^−^^1^
*c* = 24.2623 (7) Å	*T* = 290 K
β = 94.0767 (12)°	Irregular, colourless
*V* = 1894.28 (8) Å^3^	0.33 × 0.30 × 0.29 mm
*Z* = 4	

### Data collection

**Table:**

Nonius KappaCCD diffractometer	3288 independent reflections
Radiation source: sealed tube	2778 reflections with *I* > 2σ(*I*)
graphite	*R*_int_ = 0.049
CCD rotation images scans	θ_max_ = 25.0°, θ_min_ = 3.3°
Absorption correction: multi-scan (*SADABS*; Sheldrick, 1996)	*h* = −9→8
*T*_min_ = 0.82, *T*_max_ = 0.85	*k* = −11→11
12888 measured reflections	*l* = −28→26

### Refinement

**Table:**

Refinement on *F*^2^	Primary atom site location: structure-invariant direct methods
Least-squares matrix: full	Secondary atom site location: difference Fourier map
*R*[*F*^2^ > 2σ(*F*^2^)] = 0.043	Hydrogen site location: inferred from neighbouring sites
*wR*(*F*^2^) = 0.121	H-atom parameters constrained
*S* = 1.05	*w* = 1/[σ^2^(*F*_o_^2^) + (0.0558*P*)^2^ + 0.758*P*] where *P* = (*F*_o_^2^ + 2*F*_c_^2^)/3
3288 reflections	(Δ/σ)_max_ < 0.001
218 parameters	Δρ_max_ = 0.37 e Å^−^^3^
0 restraints	Δρ_min_ = −0.38 e Å^−^^3^

### Special details

**Table:**

Geometry. All s.u.'s (except the s.u. in the dihedral angle between two l.s. planes) are estimated using the full covariance matrix. The cell s.u.'s are taken into account individually in the estimation of s.u.'s in distances, angles and torsion angles; correlations between s.u.'s in cell parameters are only used when they are defined by crystal symmetry. An approximate (isotropic) treatment of cell s.u.'s is used for estimating s.u.'s involving l.s. planes.
Refinement. Refinement of *F*^2^ against ALL reflections. The weighted *R*-factor *wR* and goodness of fit *S* are based on *F*^2^, conventional *R*-factors *R* are based on *F*, with *F* set to zero for negative *F*^2^. The threshold expression of *F*^2^ > σ(*F*^2^) is used only for calculating *R*-factors(gt) *etc*. and is not relevant to the choice of reflections for refinement. *R*-factors based on *F*^2^ are statistically about twice as large as those based on *F*, and *R*- factors based on ALL data will be even larger.

### Fractional atomic coordinates and isotropic or equivalent isotropic displacement parameters (Å^2^)

**Table:**

	*x*	*y*	*z*	*U*_iso_*/*U*_eq_	
C2	0.3322 (3)	0.3195 (2)	0.66893 (9)	0.0514 (5)	
C3	0.4451 (2)	0.2265 (2)	0.70636 (9)	0.0490 (5)	
C4	0.3506 (3)	0.1218 (2)	0.73838 (9)	0.0527 (5)	
H4A	0.3189	0.0448	0.7145	0.063*	
H4B	0.4223	0.0876	0.7692	0.063*	
C5	0.1959 (3)	0.1856 (3)	0.75971 (10)	0.0629 (6)	
H5A	0.1407	0.1199	0.7823	0.075*	
H5B	0.2264	0.2650	0.7823	0.075*	
C6	0.0808 (3)	0.2277 (3)	0.71127 (11)	0.0697 (7)	
H6A	0.0309	0.1464	0.6940	0.084*	
H6B	−0.0085	0.2835	0.7243	0.084*	
C7	0.6208 (2)	0.2649 (2)	0.81264 (9)	0.0520 (5)	
C8	0.7382 (3)	0.1601 (2)	0.81591 (10)	0.0557 (5)	
H8	0.7841	0.1296	0.7840	0.067*	
C9	0.7876 (3)	0.1008 (2)	0.86615 (11)	0.0632 (6)	
H9	0.8659	0.0303	0.8681	0.076*	
C10	0.7204 (3)	0.1464 (3)	0.91293 (11)	0.0678 (7)	
C11	0.6026 (3)	0.2500 (3)	0.91080 (11)	0.0756 (7)	
H11	0.5572	0.2800	0.9429	0.091*	
C12	0.5534 (3)	0.3082 (3)	0.86065 (10)	0.0673 (7)	
H12	0.4737	0.3776	0.8589	0.081*	
C13	0.4783 (3)	0.0506 (2)	0.61552 (9)	0.0572 (5)	
C14	0.4608 (3)	−0.0901 (3)	0.61981 (11)	0.0687 (6)	
H14	0.5093	−0.1360	0.6505	0.082*	
C15	0.3712 (4)	−0.1629 (3)	0.57846 (13)	0.0814 (8)	
H15	0.3600	−0.2576	0.5811	0.098*	
C16	0.2998 (3)	−0.0938 (4)	0.53379 (12)	0.0798 (8)	
C17	0.3157 (4)	0.0450 (4)	0.52858 (12)	0.0869 (9)	
H17	0.2660	0.0900	0.4979	0.104*	
C18	0.4061 (4)	0.1180 (3)	0.56924 (11)	0.0744 (7)	
H18	0.4188	0.2123	0.5657	0.089*	
C19	0.0614 (4)	0.3812 (3)	0.62912 (13)	0.0835 (8)	
H19A	0.0725	0.4778	0.6362	0.125*	
H19B	−0.0530	0.3545	0.6314	0.125*	
H19C	0.0954	0.3612	0.5928	0.125*	
O1	0.3934 (2)	0.40242 (17)	0.63843 (7)	0.0719 (5)	
Cl1	0.78388 (14)	0.07472 (10)	0.97627 (4)	0.1129 (3)	
Cl2	0.19021 (13)	−0.18374 (14)	0.48074 (4)	0.1289 (4)	
S1	0.56494 (8)	0.35278 (6)	0.75025 (3)	0.0615 (2)	
S2	0.60276 (7)	0.14457 (7)	0.66590 (3)	0.0638 (2)	
N1	0.1666 (2)	0.3052 (2)	0.67012 (8)	0.0589 (5)	

### Atomic displacement parameters (Å^2^)

**Table:**

	*U*^11^	*U*^22^	*U*^33^	*U*^12^	*U*^13^	*U*^23^
C2	0.0504 (12)	0.0463 (11)	0.0566 (12)	−0.0034 (9)	−0.0033 (9)	−0.0035 (9)
C3	0.0412 (11)	0.0466 (11)	0.0588 (12)	−0.0035 (8)	−0.0002 (9)	−0.0038 (9)
C4	0.0553 (12)	0.0434 (11)	0.0585 (13)	−0.0066 (9)	−0.0020 (10)	−0.0008 (9)
C5	0.0609 (14)	0.0606 (13)	0.0688 (15)	−0.0116 (11)	0.0150 (11)	−0.0024 (11)
C6	0.0474 (13)	0.0693 (15)	0.0928 (19)	−0.0050 (11)	0.0079 (12)	−0.0031 (13)
C7	0.0425 (11)	0.0500 (11)	0.0621 (12)	−0.0023 (9)	−0.0056 (9)	−0.0108 (10)
C8	0.0446 (11)	0.0576 (12)	0.0646 (14)	0.0018 (9)	0.0013 (10)	−0.0100 (10)
C9	0.0493 (12)	0.0538 (13)	0.0846 (17)	0.0044 (10)	−0.0083 (11)	−0.0089 (12)
C10	0.0654 (15)	0.0707 (15)	0.0645 (15)	−0.0047 (12)	−0.0161 (12)	−0.0043 (12)
C11	0.0682 (16)	0.0962 (19)	0.0615 (15)	0.0109 (14)	−0.0027 (12)	−0.0207 (14)
C12	0.0574 (14)	0.0725 (15)	0.0699 (16)	0.0178 (12)	−0.0097 (11)	−0.0204 (12)
C13	0.0451 (11)	0.0698 (14)	0.0566 (13)	0.0102 (10)	0.0043 (9)	−0.0087 (10)
C14	0.0633 (15)	0.0689 (16)	0.0735 (16)	0.0100 (12)	0.0019 (12)	−0.0054 (12)
C15	0.0790 (19)	0.0766 (17)	0.090 (2)	−0.0058 (14)	0.0131 (15)	−0.0209 (15)
C16	0.0612 (16)	0.109 (2)	0.0690 (17)	−0.0075 (15)	0.0079 (13)	−0.0283 (16)
C17	0.086 (2)	0.112 (2)	0.0606 (16)	0.0151 (17)	−0.0100 (14)	−0.0096 (16)
C18	0.0828 (18)	0.0781 (17)	0.0609 (15)	0.0126 (14)	−0.0043 (13)	−0.0028 (12)
C19	0.0666 (17)	0.0893 (19)	0.0905 (19)	0.0192 (14)	−0.0237 (14)	−0.0028 (15)
O1	0.0693 (11)	0.0663 (10)	0.0790 (11)	−0.0096 (8)	−0.0020 (9)	0.0199 (9)
Cl1	0.1408 (8)	0.1145 (7)	0.0781 (5)	0.0100 (6)	−0.0305 (5)	0.0123 (5)
Cl2	0.1044 (7)	0.1885 (11)	0.0944 (6)	−0.0460 (7)	0.0104 (5)	−0.0639 (7)
S1	0.0640 (4)	0.0479 (3)	0.0702 (4)	−0.0118 (2)	−0.0113 (3)	−0.0013 (2)
S2	0.0407 (3)	0.0814 (4)	0.0688 (4)	0.0036 (3)	−0.0002 (3)	−0.0123 (3)
N1	0.0470 (10)	0.0599 (11)	0.0686 (12)	0.0035 (8)	−0.0055 (8)	−0.0025 (9)

### Geometric parameters (Å, °)

**Table:**

C2—O1	1.222 (3)	C10—C11	1.382 (4)
C2—N1	1.340 (3)	C10—Cl1	1.731 (3)
C2—C3	1.533 (3)	C11—C12	1.375 (4)
C3—C4	1.518 (3)	C11—H11	0.9300
C3—S2	1.839 (2)	C12—H12	0.9300
C3—S1	1.851 (2)	C13—C14	1.383 (4)
C4—C5	1.513 (3)	C13—C18	1.391 (3)
C4—H4A	0.9700	C13—S2	1.777 (2)
C4—H4B	0.9700	C14—C15	1.388 (4)
C5—C6	1.500 (3)	C14—H14	0.9300
C5—H5A	0.9700	C15—C16	1.367 (4)
C5—H5B	0.9700	C15—H15	0.9300
C6—N1	1.463 (3)	C16—C17	1.366 (4)
C6—H6A	0.9700	C16—Cl2	1.743 (3)
C6—H6B	0.9700	C17—C18	1.380 (4)
C7—C12	1.385 (3)	C17—H17	0.9300
C7—C8	1.388 (3)	C18—H18	0.9300
C7—S1	1.769 (2)	C19—N1	1.460 (3)
C8—C9	1.381 (3)	C19—H19A	0.9600
C8—H8	0.9300	C19—H19B	0.9600
C9—C10	1.365 (4)	C19—H19C	0.9600
C9—H9	0.9300		
			
O1—C2—N1	121.6 (2)	C9—C10—Cl1	119.9 (2)
O1—C2—C3	120.21 (19)	C11—C10—Cl1	119.0 (2)
N1—C2—C3	118.18 (19)	C12—C11—C10	119.2 (2)
C4—C3—C2	113.84 (17)	C12—C11—H11	120.4
C4—C3—S2	111.66 (14)	C10—C11—H11	120.4
C2—C3—S2	109.95 (14)	C11—C12—C7	120.9 (2)
C4—C3—S1	114.26 (15)	C11—C12—H12	119.5
C2—C3—S1	102.08 (13)	C7—C12—H12	119.5
S2—C3—S1	104.26 (10)	C14—C13—C18	119.3 (2)
C5—C4—C3	110.55 (17)	C14—C13—S2	120.93 (19)
C5—C4—H4A	109.5	C18—C13—S2	119.6 (2)
C3—C4—H4A	109.5	C13—C14—C15	120.2 (3)
C5—C4—H4B	109.5	C13—C14—H14	119.9
C3—C4—H4B	109.5	C15—C14—H14	119.9
H4A—C4—H4B	108.1	C16—C15—C14	119.2 (3)
C6—C5—C4	108.67 (19)	C16—C15—H15	120.4
C6—C5—H5A	110.0	C14—C15—H15	120.4
C4—C5—H5A	110.0	C17—C16—C15	121.6 (3)
C6—C5—H5B	110.0	C17—C16—Cl2	118.4 (3)
C4—C5—H5B	110.0	C15—C16—Cl2	119.9 (3)
H5A—C5—H5B	108.3	C16—C17—C18	119.5 (3)
N1—C6—C5	112.44 (19)	C16—C17—H17	120.2
N1—C6—H6A	109.1	C18—C17—H17	120.2
C5—C6—H6A	109.1	C17—C18—C13	120.1 (3)
N1—C6—H6B	109.1	C17—C18—H18	119.9
C5—C6—H6B	109.1	C13—C18—H18	119.9
H6A—C6—H6B	107.8	N1—C19—H19A	109.5
C12—C7—C8	118.7 (2)	N1—C19—H19B	109.5
C12—C7—S1	118.79 (17)	H19A—C19—H19B	109.5
C8—C7—S1	122.34 (17)	N1—C19—H19C	109.5
C9—C8—C7	120.6 (2)	H19A—C19—H19C	109.5
C9—C8—H8	119.7	H19B—C19—H19C	109.5
C7—C8—H8	119.7	C7—S1—C3	105.06 (10)
C10—C9—C8	119.5 (2)	C13—S2—C3	102.48 (9)
C10—C9—H9	120.3	C2—N1—C19	117.4 (2)
C8—C9—H9	120.3	C2—N1—C6	125.80 (19)
C9—C10—C11	121.1 (2)	C19—N1—C6	116.7 (2)
			
O1—C2—C3—C4	−175.5 (2)	C14—C15—C16—C17	0.6 (4)
N1—C2—C3—C4	3.4 (3)	C14—C15—C16—Cl2	178.7 (2)
O1—C2—C3—S2	−49.4 (2)	C15—C16—C17—C18	0.0 (5)
N1—C2—C3—S2	129.55 (18)	Cl2—C16—C17—C18	−178.1 (2)
O1—C2—C3—S1	60.9 (2)	C16—C17—C18—C13	−0.9 (4)
N1—C2—C3—S1	−120.22 (18)	C14—C13—C18—C17	1.1 (4)
C2—C3—C4—C5	−40.9 (2)	S2—C13—C18—C17	177.0 (2)
S2—C3—C4—C5	−166.11 (15)	C12—C7—S1—C3	−113.91 (19)
S1—C3—C4—C5	75.9 (2)	C8—C7—S1—C3	70.55 (19)
C3—C4—C5—C6	63.6 (2)	C4—C3—S1—C7	29.71 (18)
C4—C5—C6—N1	−48.4 (3)	C2—C3—S1—C7	153.07 (14)
C12—C7—C8—C9	−0.4 (3)	S2—C3—S1—C7	−92.45 (12)
S1—C7—C8—C9	175.18 (17)	C14—C13—S2—C3	−104.0 (2)
C7—C8—C9—C10	−0.3 (3)	C18—C13—S2—C3	80.2 (2)
C8—C9—C10—C11	0.6 (4)	C4—C3—S2—C13	67.21 (17)
C8—C9—C10—Cl1	−178.95 (18)	C2—C3—S2—C13	−60.15 (16)
C9—C10—C11—C12	−0.3 (4)	S1—C3—S2—C13	−168.93 (11)
Cl1—C10—C11—C12	179.3 (2)	O1—C2—N1—C19	6.9 (3)
C10—C11—C12—C7	−0.3 (4)	C3—C2—N1—C19	−172.0 (2)
C8—C7—C12—C11	0.7 (4)	O1—C2—N1—C6	−168.6 (2)
S1—C7—C12—C11	−175.0 (2)	C3—C2—N1—C6	12.5 (3)
C18—C13—C14—C15	−0.4 (4)	C5—C6—N1—C2	11.0 (3)
S2—C13—C14—C15	−176.2 (2)	C5—C6—N1—C19	−164.5 (2)
C13—C14—C15—C16	−0.5 (4)		

### Hydrogen-bond geometry (Å, °)

**Table:**

Cg1 is the centroid of the C7–C12.

**Table:**

*D*—H···*A*	*D*—H	H···*A*	*D*···*A*	*D*—H···*A*
C9—H9···O1^i^	0.93	2.32	3.218 (3)	164
C11—H11···Cl2^ii^	0.93	2.83	3.708 (3)	157
C19—H19a···Cg1^iii^	0.96	2.95	3.676 (3)	133

Symmetry codes: (i) −*x*+3/2, *y*−1/2, −*z*+3/2; (ii) −*x*+1/2, *y*+1/2, −*z*+3/2; (iii) −*x*+1/2, *y*−1/2, −*z*+3/2.
